# The imperative of digital competence in healthcare professionals: comparison between the North and South of Europe

**DOI:** 10.1186/s12913-026-14000-8

**Published:** 2026-01-10

**Authors:** Marco Esperança, Joao C. Ferreira, Ana Lucia Martins

**Affiliations:** 1 Instituto Universitario de Lisboa ISCTE-IUL, Lisbon, 1649-026 Portugal; 2 Faculty of Logistics, Molde University College, Molde, NO-6410 Norway; 3 INESC INOV-Lab, Lisbon, 1000-029 Portugal; 4 Business Research Unit (BRU-IUL), Iscte-University Institute of Lisbon (ISCTE-IUL), Lisbon, 1649-026 Portugal

**Keywords:** Digital competencies, Digital health, Healthcare workforce training, Digital transformation

## Abstract

**Background:**

Digital transformation in healthcare increasingly depends on the digitalisation of day-to-day clinical work, in particular on professionals’ ability to use electronic health records, telemedicine, and data-driven tools. However, gaps in digital competence and uneven access to training persist across Europe. This study examines how healthcare professionals understand and apply digital tools in clinical practice, and compares perceived barriers between Northern and Southern Europe.

**Methods:**

We conducted a cross-sectional qualitative survey using a digital self-assessment questionnaire administered to 2,048 healthcare professionals from six European countries. The study was theoretically grounded in the European Digital Competence Framework for Citizens 2.0 (DigComp 2.0) and the eHealth Literacy Framework, and employed thematic analysis to interpret open-ended responses.

**Results:**

Four key thematic areas emerged: remote patient care and monitoring; digital education and health management; transformation of clinical workflows; and data-driven diagnostics. Professionals in Northern Europe—especially Finland—described integrated digital systems, structured training, and institutional support. Participants in Southern Europe more often reported fragmented infrastructure, limited training opportunities, and organizational resistance. The most frequently cited barriers to digital adoption were lack of training (23%), time constraints (27%), limited resources (24%), and resistance to change (19%).

**Conclusions:**

Healthcare professionals widely view digital competence as essential for safe and effective care, but uptake is constrained by structural barriers and regional inequalities. Targeted investment in workforce training, protected time for skill development, and foundational digital infrastructure—particularly in Southern Europe and in resource-limited settings—is needed to support equitable digital transformation across European health systems.

**Supplementary Information:**

The online version contains supplementary material available at 10.1186/s12913-026-14000-8.

## Background

Digital technologies such as electronic health records (EHRs), telemedicine, artificial intelligence (AI), and data analytics are now embedded in routine healthcare delivery. These tools shape clinical workflows, information exchange, decision-making, and even the safety and continuity of care. Digital competence — the ability of healthcare professionals to confidently and appropriately use these tools in practice — is directly linked to care quality and patient safety, because accurate documentation, secure information exchange, and appropriate use of decision-support tools depend on clinicians’ ability to work effectively with digital systems. In this sense, digital competence has become a core component of safe, high-quality, and efficient healthcare, rather than an optional technical skill [1–4].

In this article, we distinguish between digitalisation and digital transformation. We use *digitalisation* to refer to the concrete integration and routine use of digital technologies in clinical work—for example, electronic health records, telemedicine platforms, remote monitoring systems, and digital patient management tools. We use *digital transformation* to denote the broader, system-level changes in service delivery models, governance, workflows, and professional roles that are enabled by digitalisation at scale. Our empirical analysis focuses primarily on how healthcare professionals experience the digitalisation of clinical practice, while the Discussion and Conclusion situate these experiences within the wider digital transformation of European health systems. At the same time, health systems increasingly view workforce-wide digital capability as critical for service accessibility, coordination, and sustainability. Recent European policy discussions reflect this by treating digital competence as a requirement for workforce readiness and patient safety, not only as an innovation goal [3, 5].

However, developing and applying digital competence in practice remains challenging for many healthcare professionals. Reported barriers include lack of time for training, limited institutional support, concerns about data security and interoperability, fragmented systems, and uneven access to formal digital training opportunities [1, 6–10]. These barriers are not only organizational irritants; they directly affect clinicians’ ability to document accurately, coordinate care, and use decision-support tools in ways that benefit patients. In other words, deficits in digital competence can translate into inefficiencies, safety risks, and inequities in care delivery [1, 2, 4, 11].

Although the COVID-19 pandemic accelerated the uptake of telehealth, remote monitoring, and digital communication tools across Europe, it also exposed gaps in digital readiness. Many services were forced to digitalize quickly, but not all healthcare professionals felt prepared to work in that environment [9, 12, 13]. It is important to note that, while this broader context of accelerated digitalisation informed the motivation for the present study, the questionnaire used here did not include explicit COVID-19–specific items. We address this as a limitation in the Discussion.

Existing work has described common barriers to digital adoption, such as high workload, insufficient infrastructure, and resistance to change within organisations [8, 10, 14, 15]. Other studies have highlighted disparities in digital skills across professional groups and demographic subgroups, often noting higher confidence among younger professionals or those with more recent training [11, 16, 17]. Still others have argued for the need to pair technical skills (use of software, data entry, device handling) with soft skills such as communication, critical thinking, and empathy to ensure that digitalisation supports, rather than undermines, the human relationship with patients [18]. Collectively, this literature suggests that digital competence is multidimensional: it spans technical proficiency, information management, ethical and safety awareness, and the ability to integrate digital tools into clinical, managerial, and interpersonal aspects of care [2, 6, 14, 18].

Despite increasing attention to digitalisation in healthcare, there remains limited comparative evidence on how these competencies and challenges are experienced across different parts of Europe. Prior work often focuses on single countries, specific professions, or specific technologies [2, 19, 20]. Far fewer studies have compared healthcare professionals’ perceptions of digital readiness, barriers, and support structures across regions with very different infrastructures, funding models, and policy environments. This comparative perspective matters. Healthcare systems in Northern Europe are frequently described as digitally mature, with integrated national platforms and institutionalised training pathways, whereas systems in parts of Southern Europe face more fragmented digital infrastructures and more variable organisational support [21–23]. Understanding whether, and how, these structural differences are experienced by frontline staff is essential for designing targeted workforce development strategies and for informing digital health policy.

Against this background, and recognising that digitalisation is not experienced uniformly across Europe, this study focuses on how healthcare professionals describe their digital competence, access to training, and the conditions that enable or hinder the use of digital tools in practice. The primary aim of the study is to characterise the digital competence landscape among healthcare professionals across several European countries, identify key barriers to the effective use of digital tools in clinical practice, and assess how training opportunities and infrastructural support differ between Northern and Southern Europe.

To operationalise this aim, we address the following research questions (RQ):

### RQ1

What are the main barriers healthcare professionals face in developing and applying digital competencies in clinical practice?

### RQ2

How do digital training opportunities, infrastructure, and institutional support vary between Northern and Southern European healthcare systems?

### RQ3

What regional patterns can be identified in digital health readiness and perceived competence among healthcare professionals?

The theoretical understanding of digital competence and technology uptake among healthcare professionals can be framed through established adoption models such as the Technology Acceptance Model (TAM) and the Unified Theory of Acceptance and Use of Technology (UTAUT) [24, 25]. TAM posits that technology use is primarily driven by perceived usefulness and perceived ease of use, which together shape users’ behavioral intentions and actual adoption [25]. UTAUT extends this model by integrating constructs such as performance expectancy, effort expectancy, social influence, and facilitating conditions—factors highly relevant to healthcare environments where institutional support, workload pressures, and interoperability issues affect adoption [24]. Applying these frameworks helps explain why even digitally skilled professionals may struggle to integrate new systems when organisational or contextual enablers are weak. In this study, these models inform the interpretation of barriers and facilitators of digital competence across Europe, complementing the DigComp 2.0 framework’s multidimensional perspective on digital skills [26].

## Methodology

### Data collection and analysis

We conducted a cross-sectional qualitative survey of healthcare professionals in Portugal, Greece, Italy, Finland, Norway, and Denmark to compare digital competence, perceived readiness, and barriers to digital transformation between Northern and Southern Europe. Eligible participants were physicians, nurses, radiographers, IT specialists in clinical support, and departmental or clinical administrators in hospital or primary care settings. Inclusion criteria were current practice in one of the six countries, routine use of at least one digital health tool (for example electronic health records, telemedicine, remote monitoring, digital patient management), and ability to respond in English. Recruitment was carried out through institutional mailing lists and professional networks (clinical departments, national associations, clinical IT units). A total of 4,950 invitations were sent, and 2,048 professionals completed the questionnaire between 1 December 2024 and 15 January 2025 (completion rate 41.4%). No incentives were offered. The study received ethical approval from the Ethics Committee of the School of Technologies and Architecture of ISCTE (CE-ISTA/2025.04). Participants provided digital informed consent, and no identifying data (including IP address) were collected. Data were collected using the AMR Educare platform, which in this study functioned only as a secure survey host and response capture system [25]. The questionnaire was developed by the research team and was not related to antimicrobial resistance. It contained 20 open-ended prompts in four areas: use of digital tools; training experiences and needs; perceived barriers and organisational support; and attitudes toward digital transformation in daily clinical work. These areas reflect the European Commission’s Digital Competence Framework for Citizens 2.0 (DigComp 2.0), including information and data literacy, communication and collaboration, content creation, safety, and problem solving [8, 26]. The instrument was reviewed by five experts in digital health, clinical education, and health systems organisation, pilot-tested with 50 professionals across roles and countries, and refined for clarity. Because all questions were open-ended rather than scale-based, no psychometric reliability statistics were calculated.

All responses were exported from AMR Educare and processed in Python (Pandas, NLTK, spaCy). Preprocessing included lowercasing, removal of punctuation and stop words, lemmatisation, and standardisation of technical terms (for example mapping “electronic health record,” “EHR,” and “patient file system” to a single label). We then generated word frequency and co-occurrence summaries to identify recurring areas (for example training, time pressure, infrastructure, security) and to surface less frequent but important issues (for example resistance to change, bandwidth limitations). These computational summaries were used only to guide coding and were not treated as findings [8]. We then conducted inductive thematic analysis in NVivo following Braun and Clarke’s six-phase approach [24]. Two researchers independently coded a stratified subsample of 200 responses (balanced by country and role), reconciled differences, and produced a shared codebook, which was then applied to the full dataset. Approximately 20% of responses were double-coded; disagreements were resolved by consensus, and code definitions were refined iteratively. Because coding evolved during analysis, we did not calculate a fixed inter-coder kappa; consistency was maintained through discussion and coder memos.

Coded material was grouped into higher-order categories (for example time constraints, access to training, infrastructure and support, organisational resistance, workflow disruption, data governance). These categories were then organised around the three research questions (barriers to competence, regional variation, and perceived confidence) for reporting in the Results. These domains align with DigComp 2.0 (information and data literacy, communication and collaboration, safety, problem solving) and with technology adoption models such as the TAM and the UTAUT [24–27]. For each barrier or enabler, we calculated the proportion of respondents who explicitly mentioned it (for example time constraints, lack of training, limited resources, resistance to change) and compared Northern and Southern Europe using Pearson’s chi-square tests (SciPy, Python) for selected coded themes such as access to training and routine digital tool use. For regional analysis, Portugal (*n* = 500), Greece (*n* = 200), and Italy (*n* = 324) were grouped as Southern Europe; Finland (*n* = 424), Norway (*n* = 330), and Denmark (*n* = 270) were grouped as Northern Europe. Each macro-region contributed 1,024 respondents. This balance reflects predefined recruitment targets rather than national workforce proportions.

These six countries were purposefully selected to represent two contrasting European contexts in terms of digital health maturity and workforce support. The Nordic countries in our sample (Finland, Norway, Denmark) are repeatedly characterised in EU and WHO Europe assessments as digitally mature health systems, with nationally integrated electronic health records, interoperable data infrastructures, and established frameworks for secondary use of health and social data (for example Finland’s Kanta system), alongside high levels of population-level digital skills and structured digital training pathways for healthcare staff [28, 29]. By contrast, Southern European health systems such as Portugal, Greece, and Italy are described in EU monitoring and audit reports as still facing fragmentation, variable interoperability, uneven connectivity (especially in rural settings), and less consistent institutional support for workforce digital upskilling; more broadly, EU analyses continue to document a North–South divide in digital readiness and infrastructure [30, 31]. Grouping them as “Northern” and “Southern” regions therefore enables meaningful comparison between digitally mature and less mature healthcare environments, consistent with prior European digital health literature [21–23].

**Fig. 1:**
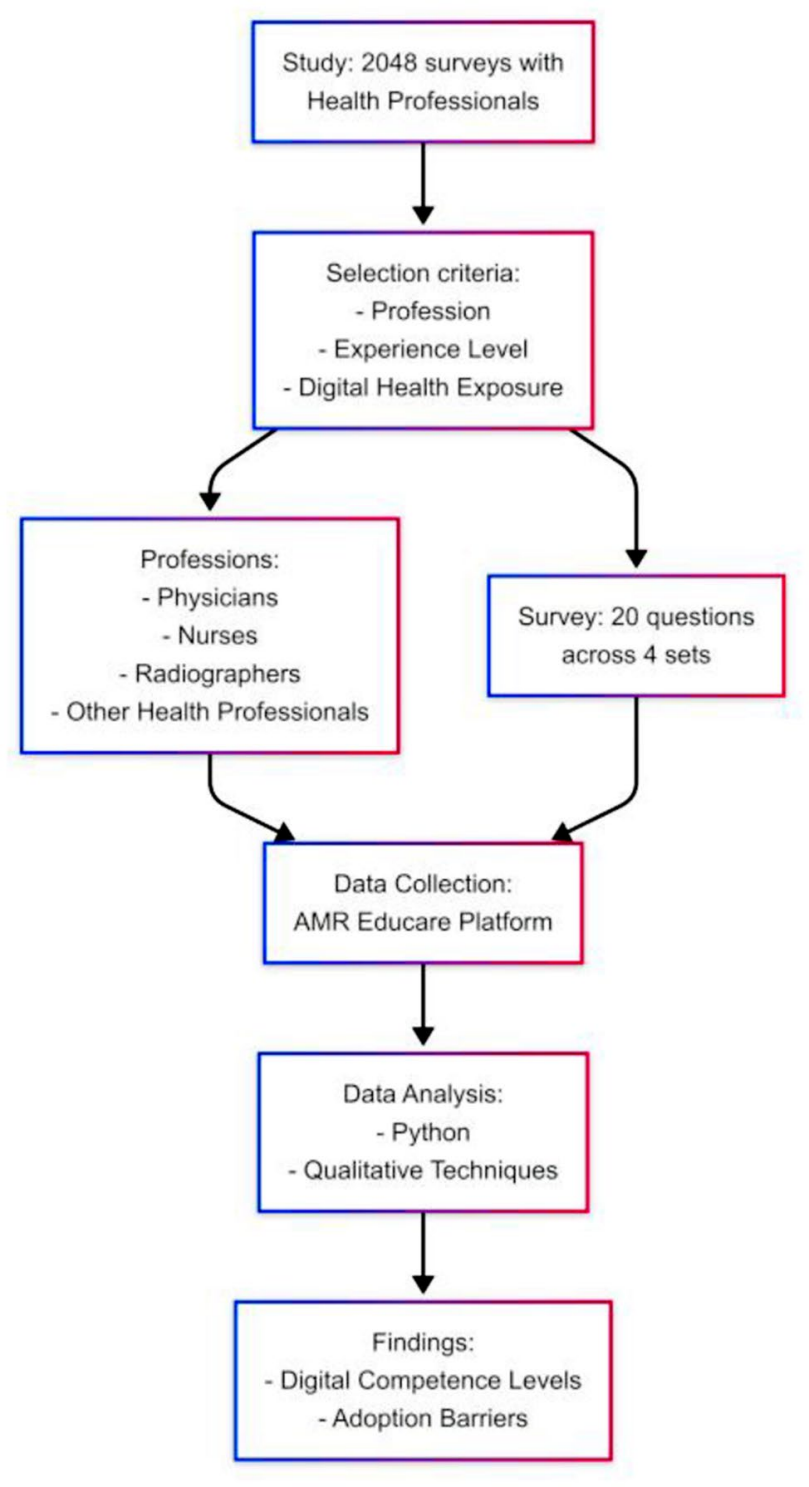
Workflow adopted in the study

This mixed-method analysis approach—grounded in CRISP-DM and Braun & Clarke’s thematic analysis—provided both structure and flexibility in deriving insights from a large and diverse corpus of qualitative responses. Figure 1 presents the workflow adopted in the study:

### Study population

Participants in this study were selected based on multiple criteria, including their professional roles (physicians, nurses, radiographers, IT specialists, and departmental administrators), levels of experience (early-career, mid-level, and experienced professionals), and familiarity with the digital health systems under investigation. Preference was given to individuals with prior exposure to digital health tools, as their insights were considered particularly relevant to the study’s objectives. The selection process also aimed to ensure representation from a variety of healthcare settings—urban and rural, as well as public and private institutions—in order to capture a broad spectrum of challenges and opportunities.

The study population consisted of 2048 healthcare professionals working in hospitals and primary care centers across the participating countries. The sample included 30% nurses, 25% physicians, 15% radiographers, 20% IT specialists, and 10% departmental administrators. In terms of experience, 20% of participants were early-career professionals, 35% were at a mid-career level, and 45% were experienced professionals. Additionally, 60% of respondents were based in urban settings, while 40% worked in rural areas. This distribution ensured a diverse and representative sample, supporting the study’s aim to comprehensively assess digital health systems from multiple professional and contextual perspectives. Tables 1 and 2 present the characterization of the study population:

**Table 1:** Characterization of participants by experience level

Experience Level	Description	Number of participants	Percentage of participants
Early Career Professionals	Recent graduates and entry-level practitioners with < 2 years	406	20%
Mid-level Professionals	Professionals with 2 to 5 years of experience	710	35%
Experienced Professionals	Senior professionals with over 5 years of experience	932	45%

**Table 2:** Distribution of participants by country

Country	Number of participants		Percentage of participants
Portugal	500		24%
Greece	200		10%
Italy	324		16%
Finland	424		21%
Norway	330		16%
Denmark	270		13%

As we can see, we have 1,024 participants in each region — Northern Europe and Southern Europe.

## Results

To align the analysis with the study aims and with existing implementation literature on digitalisation in healthcare, we present the findings according to the three research questions (RQ1–RQ3). For RQ1, we organise reported barriers into four widely described determinants of digital adoption in clinical settings: (i) workload and time constraints, which limit opportunities for training and safe experimentation with new systems [12, 14, 22]; (ii) gaps in access to structured digital skills training and ongoing support [3, 5, 10]; (iii) infrastructural and interoperability limitations, including unreliable connectivity, fragmented records, and insufficient technical support [29, 31]; and (iv) organisational resistance or low perceived value of new tools, often linked to poor workflow integration and change management [9, 12, 22]. RQ2 focuses on regional differences between Northern and Southern Europe in these same determinants. RQ3 addresses perceived confidence and digital readiness at the individual level, particularly in relation to data use and decision support. This structure allows us to locate our findings within the current state of the literature on digital competence and digital transformation in healthcare while directly answering the research questions.

### RQ1: Barriers to digital competence

Most respondents reported that digital tools are already part of routine practice. 67% described regular use of electronic health records, telemedicine platforms, remote monitoring systems, or digital patient management systems. In addition, 85% stated that digital competence is essential for delivering safe, efficient care. As one participant noted, “Digital systems are no longer optional. If you can’t use them, you can’t work effectively with patients anymore” (Nurse, Finland).

Despite this recognition, respondents identified several barriers that limit their ability to develop and apply digital skills. 23% reported having received little or no formal training in digital health technologies. One administrator from Greece stated, “We are expected to use new systems, but no training is provided beyond basic manuals.” A nurse from Denmark similarly commented, “There’s simply no time for training in a regular shift.”

Time pressure was the single most frequently reported barrier. 27% of participants described workload and lack of protected time as obstacles to learning and integrating new digital tools. A mid-career physician in Italy wrote, “We barely keep up with clinical work. There is no realistic window to learn a new platform properly.”

Resource constraints were also widely reported. 24% of respondents cited limited infrastructure, outdated hardware, poor connectivity, or insufficient IT support as barriers to digital adoption. This was particularly visible in rural and resource-constrained settings. One nurse in Portugal explained, “Telehealth is transformative, but our hospital lacks the internet bandwidth to support it.” A physician in Italy described still relying on “faxed data” for remote monitoring.

Resistance to change within organisations was identified by 19% of participants. This included reluctance among colleagues to abandon established paper-based workflows, concern about data security, and scepticism about the practical value of new systems. A senior administrator in Norway noted, “People don’t refuse outright, but they find reasons not to use the new tools. They say it’s unsafe, or too complicated, or ‘not how we do things here.’”.

**Fig. 2:**
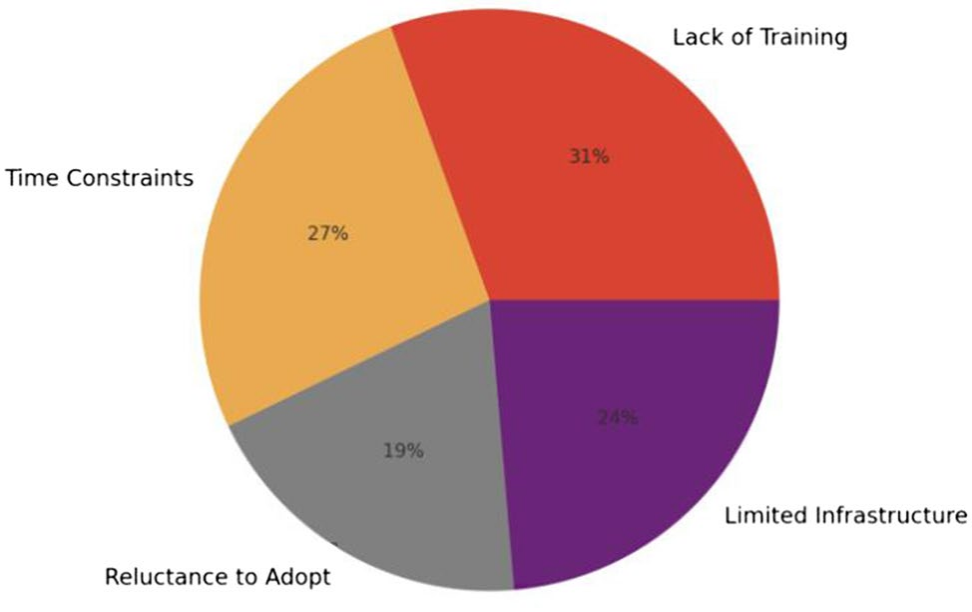
Main barrier to digital adoption

Figure 2 summarises the most commonly cited barriers to digital adoption: time constraints (27%), limited resources/infrastructure (24%), lack of training (23%), and resistance to change (19%).

### RQ2: Regional variation in training and infrastructure

Marked regional differences emerged between Northern and Southern Europe in both access to training and ability to apply digital tools in practice. Only 49% of respondents in Southern Europe (Portugal, Greece, Italy) reported having access to structured training in digital systems (for example formal onboarding, continuing education, simulation-based training). In contrast, 81% of respondents in Northern Europe (Finland, Norway, Denmark) reported such access. This difference was statistically significant (χ² = 45.67, *p* < 0.001).

Use of digital tools was also more embedded in the North. 91% of respondents in Northern Europe reported frequent or routine use of digital systems in daily work, compared to 56% in Southern Europe. Respondents in Finland repeatedly mentioned national infrastructure such as the Kanta system as an enabler of consistent digital practice. One Finnish nurse commented, “Everything is connected. I can see medication history, lab results, referrals in one place. It saves time and avoids mistakes.” By contrast, an Italian physician described fragmented systems and workaround-heavy workflows: “Remote monitoring would help our patients, but our unit still relies on faxed data.”

**Fig. 3:**
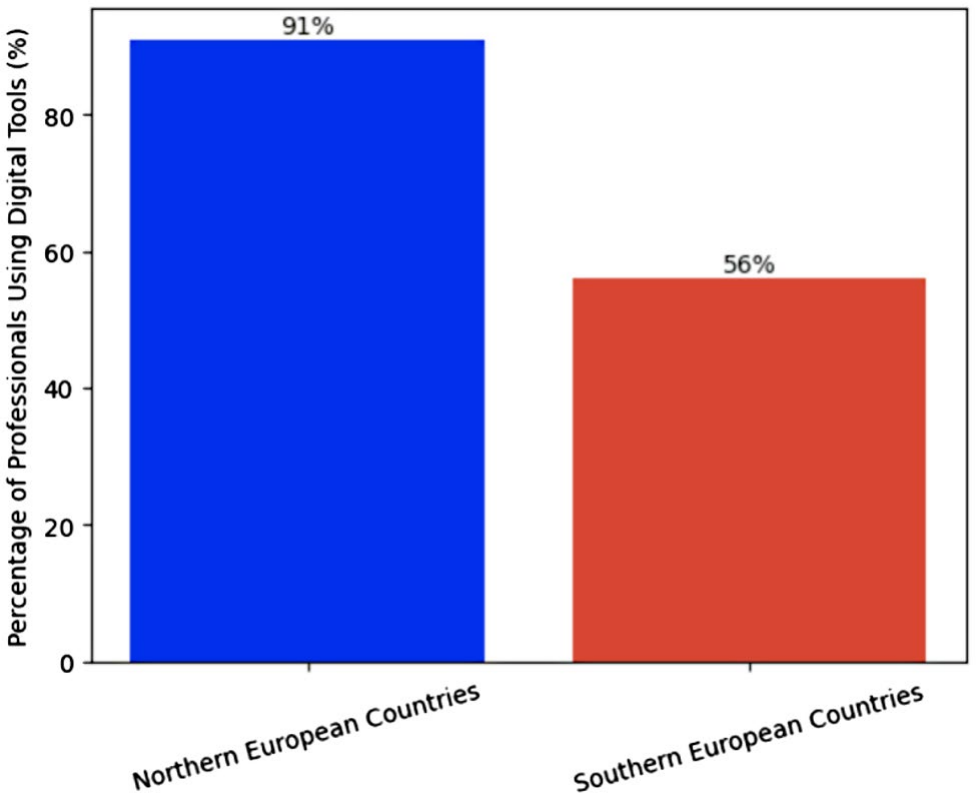
Regional differences in digital adoption

Figure 3 shows this regional divide: nearly all respondents in Northern Europe reported frequent use of digital tools (91%), compared with just over half in Southern Europe (56%).

Infrastructure and technical support were frequently cited as structural explanations for these differences. Poor connectivity, lack of interoperable records, and outdated equipment were described far more often by respondents in Southern and rural settings than by those in Northern and urban settings. A nurse in Portugal described “trying to run teleconsultations on a connection that drops every few minutes.” A radiographer in Greece noted, “The equipment is there in theory, but half the time it’s down, and there’s no one to fix it.”

Figure 3 highlights regional differences in digital adoption, with 91% of professionals in Northern Europe reporting frequent tool use compared to 56% in Southern Europe.

### RQ3: Patterns in perceived competence

While 85% of respondents agreed that digital competence improves both care quality and workflow efficiency, there were substantial regional and contextual differences in how confident professionals felt using digital systems.

Confidence in using data-driven tools (for example dashboards, analytics views, clinical decision support) was higher in Northern Europe. In Finland, 83% of respondents reported feeling confident or very confident in interpreting digital dashboards and using electronic data to support clinical decisions. In Southern European countries such as Portugal and Greece, only 62% reported similar confidence levels. One physician in Greece commented, “I can interpret basic charts, but when it comes to dashboards or analytics, I’m lost.”

Professionals linked confidence directly to training and system maturity. Respondents who reported having received formal digital training also reported being 35% more efficient in managing patient data and electronic health records compared to those without such training. A nurse in Finland described this effect concretely: “With proper training, managing patient records became faster and more accurate. I spend less time searching and more time with patients.”

Confidence was consistently lower in rural and underserved settings, regardless of country. Respondents in those settings described struggling with complex interfaces, limited local support, and unstable infrastructure. A rural clinician in Norway wrote, “If something stops working mid-consultation, I have to improvise. There’s no on-site support after hours.” These accounts suggest that confidence is shaped not only by individual skill but by local organisational and technical capacity.

## Discussion

This study examined how healthcare professionals across six European countries experience, develop, and apply digital competence in clinical practice. We focused on three research questions: barriers to digital competence (RQ1), regional variation in training and infrastructure (RQ2), and patterns in self-reported digital confidence and readiness (RQ3). The discussion integrates these findings, reflects on implications for systems and policy, and acknowledges key limitations.

### Interpretation of findings in relation to RQ1–RQ3

RQ1 asked what barriers healthcare professionals face in developing and applying digital competencies. The findings show a clear mismatch between expectation and support. Most respondents described digital tools as essential to modern clinical practice, and 67% reported using electronic health records, telemedicine, remote monitoring systems, or digital patient management systems in routine work. However, 23% reported having received little or no formal training, 27% cited lack of time for training and experimentation with new systems, 24% pointed to limited resources or infrastructure, and 19% described resistance to change within their organisations. These barriers are not purely individual; they are embedded in workload structures, institutional priorities, and infrastructure [8, 9, 15]. A nurse in Denmark observed, “There’s simply no time for training in a regular shift,” and an administrator in Greece stated, “We are expected to use new systems, but no training is provided beyond basic manuals.” These statements reflect two interlinked pressures: an assumption that digital competence is “baseline,” and an absence of protected conditions to build or maintain that competence [3, 6].

These results are consistent with DigComp 2.0, which treats digital competence as a set of learnable and maintainable skills, not an innate trait, and with technology acceptance models such as TAM and UTAUT, which highlight effort expectancy, facilitating conditions, and perceived ease of use as determinants of actual adoption [22–27]. When time, infrastructure, and training are not provided, digitalisation is experienced as added burden rather than as support. This helps explain why almost one in five respondents explicitly mentioned organisational reluctance or resistance to change. In practice, “resistance” in our data was not framed only as cultural conservatism, but as a response to poorly supported rollouts: “People don’t refuse outright, but they find reasons not to use the new tools… it’s too complicated, or ‘not how we do things here’,” reported a senior administrator in Norway.

RQ2 asked how training access, infrastructure, and institutional support differ between Northern and Southern Europe. Here, the contrast is striking. Professionals in Northern Europe reported broader access to structured training (81%) than those in Southern Europe (49%), and more frequent day-to-day use of digital tools (91% vs. 56%). Respondents in Finland repeatedly cited integrated national infrastructure (for example, Kanta) as enabling safe, reliable, and routine digital practice: “Everything is connected. I can see medication history, lab results, referrals in one place. It saves time and avoids mistakes.” By contrast, clinicians in Southern Europe often described fragmented systems: “Remote monitoring would help our patients, but our unit still relies on faxed data,” reported a physician in Italy. Poor connectivity and limited technical support were common in rural and underserved settings regardless of region, but these constraints were described more frequently and more urgently in the South. These findings suggest that unequal digital readiness across Europe is not only about individual skills; it is also about system maturity, interoperability, and investment. This has direct implications for equity, because it means that digital transformation currently risks amplifying existing geographic divides in access and quality [5, 22, 23].

RQ3 asked how professionals perceive their own digital competence and confidence. Although 85% of respondents agreed that digital competence improves care quality and efficiency, confidence in using more advanced tools (for example analytics dashboards or decision-support interfaces) was not evenly distributed. In Finland, 83% of respondents described themselves as confident in working with data-driven tools. In Southern countries such as Portugal and Greece, the comparable figure was 62%. Self-reported confidence was closely linked to both training and system usability [3, 6]. Respondents who reported having completed formal digital training described being 35% more efficient in managing digital records and patient data. One nurse in Finland explained, “With proper training, managing patient records became faster and more accurate. I spend less time searching and more time with patients.” In contrast, a physician in Greece said, “I can interpret basic charts, but when it comes to dashboards or analytics, I’m lost.”

Confidence also varied by practice environment. Respondents in rural or resource-constrained settings described lower confidence and higher uncertainty, even in countries that otherwise reported high overall digital maturity. One clinician noted, “If something stops working mid-consultation, I have to improvise. There’s no on-site support after hours.” This suggests that digital competence is not just a personal attribute; it is co-produced by training, infrastructure, workflow design, and local support models [15, 23, 27].

Together, these findings indicate that European healthcare professionals broadly understand digital competence as fundamental to safe and effective care, but operate under very different structural conditions. The data point to a layered divide: between North and South, between urban and rural settings, and between professionals who receive structured training and those who are expected to “figure it out” informally. These differences directly address RQ1–RQ3 and show that digital transformation is experienced not as a uniform process, but as contingent on context [1, 8, 22].

### Practical implications for health systems and policy

The study identifies several implications for workforce development, organisational governance, and European digital health policy.

First, training access is not optional. Respondents repeatedly described being required to use digital systems without adequate preparation. This is especially visible in Southern Europe, where only 49% reported access to structured training. The finding that training is associated with a 35% increase in perceived efficiency in managing records and patient data is not just educationally relevant; it is operationally relevant. Health systems that want safer documentation, fewer transcription errors, and faster information retrieval cannot treat training as an afterthought or an individual responsibility. A targeted digital skills curriculum, integrated into continuing professional development and delivered with protected time, should be considered part of core clinical governance, not a discretionary activity [2, 3, 6, 9, 15].

Second, digital rollout without workflow redesign produces resistance that is organisational rather than individual. Participants did not simply say “people don’t want change.” They described overloaded environments in which “there’s simply no time for training in a regular shift” (nurse, Denmark) and “we still rely on faxed data” (physician, Italy). This implies that adoption barriers are structural. Health organisations should explicitly resource change management: not just deploying platforms, but aligning staffing, documentation procedures, escalation pathways, and IT support. This is consistent with UTAUT’s emphasis on facilitating conditions, and with DigComp 2.0’s framing of competence as dependent on environment, not just on the worker [22, 24–27].

Third, current digital transformation risks widening regional and intra-national inequalities. Respondents in Finland described interoperable national systems as saving time and improving safety, while respondents in Portugal, Greece, and Italy described bandwidth limitations, duplicate data entry, and fragmented systems. If policy continues to assume that “digital” is intrinsically efficiency-enhancing, without investing in infrastructure in lower-resourced settings, then digitalisation may intensify rather than reduce workload. At a European level, this supports the case for coordinated investment in baseline digital infrastructure (connectivity, interoperable records, secure data exchange) as a matter of health equity. It also argues for incentives that specifically support rural and underserved contexts, where confidence was lowest and support most limited [4, 5, 23].

Fourth, confidence with data-driven tools (dashboards, analytics, decision support) is emerging as a new boundary of competence. Clinical staff in Northern Europe, especially Finland, more often described using dashboards to interpret longitudinal data and support clinical decisions. Staff in Southern Europe were more likely to describe these tools as opaque or overwhelming. As clinical decision-making becomes more data-mediated, this competence gap translates directly into differences in perceived diagnostic confidence, perceived safety, and professional autonomy. European-level digital health strategies should therefore address not only basic digital literacy, but also practical data literacy: how to interpret risk scores, triage dashboards, and trend visualisations in a clinically meaningful way. This extends DigComp 2.0’s “information and data literacy” and “problem solving” domains into routine clinical safety [1, 10, 11, 26, 27],.

Finally, the results support the idea that digital competence is not a purely “technical” skill set but includes communication, accountability, and patient-facing judgement. Professionals frequently linked digital systems to patient trust, continuity of care, and perceived quality (“It saves time and avoids mistakes,” nurse, Finland; “screen fatigue,” clinician, Southern Europe). This implies that digital competence training should not be limited to navigation of software, but should also cover patient communication in telemedicine, safe remote monitoring, and the ethical handling of clinical data. This is aligned with DigComp 2.0’s safety domain, but most respondents indicated they had not received meaningful preparation in this area [7, 8, 12, 18, 20, 26].

### Limitations

Several limitations must be considered when interpreting these findings.

First, the study relies on a structured but non-random sample. Participants were recruited through institutional mailing lists and professional networks and may be more digitally engaged, more motivated, or more opinionated about digital health than non-respondents. The overall completion rate was 41.4%. The results therefore reflect the views of active practitioners with direct exposure to digital systems, but they should not be assumed to represent entire national workforces [9, 10, 17].

Second, all data were collected through open-ended self-report. This approach allowed respondents to describe specific experiences (for example lack of bandwidth, duplicate documentation, lack of protected time), but it also introduces two biases. Respondents may over- or understate their competence, and they may selectively recall negative or disruptive experiences. We attempted to address this by combining qualitative thematic analysis with basic quantification of how often particular barriers and enablers were mentioned, and by comparing themes across regions. However, we did not observe practice directly [2, 8, 12].

Third, although we intentionally balanced the Northern and Southern subsamples (1,024 respondents each) to enable regional comparison, this does not reflect the actual size or composition of national health workforces. The results therefore allow structured comparison between regions but should not be interpreted as proportional estimates at country level [5, 21, 23].

Fourth, because the instrument was open-ended and did not include standardised competence scales, we cannot claim to have “measured” digital competence in a psychometric sense. Instead, we report perceived competence, perceived readiness, and described organisational conditions. This is appropriate for exploratory work and systems analysis, but it limits comparability with studies that use formal scoring frameworks [1, 6, 11].

### Implications and future directions

The findings point to several concrete actions for organisations and policymakers.

At the institutional level, digital training should be embedded into continuing professional development with protected time, not offered informally or ad hoc [8, 9]. Organisations that expect clinicians to use digital systems for documentation, decision support, and remote care should treat digital competence as mandatory and resourced, in the same way that they treat infection control or medication safety [2, 14, 15]. The association between training access and both confidence and perceived efficiency argues that training is a patient safety intervention, not just a professional development benefit [3, 8, 10].

At the regional and national level, health systems should align technology deployment with workflow redesign and IT support. Resistance to change in this study was often a response to poor implementation rather than a rejection of digital care. Policies that aim to accelerate digital transformation without addressing staffing, protected training time, infrastructure, and escalation pathways risk increasing frustration and eroding trust in digital systems.

At the European level, the results support coordinated investment to reduce structural gaps between Northern and Southern Europe and between urban and rural settings [12, 14, 22]. Respondents in Northern Europe described integrated, interoperable platforms; respondents in Southern Europe frequently described fragmentation, manual workarounds, and bandwidth limitations. Without targeted infrastructure investment and shared standards for interoperability, digital transformation may reinforce existing inequalities in access and quality. Policy instruments that frame digitalisation as a means of improving health system resilience must therefore include enforceable expectations for interoperability, support for rural connectivity, and incentives for consistent digital training across countries [1, 7, 13].

Finally, future research should build on two areas highlighted by the data. The first is data literacy in clinical decision-making: the ability to interpret dashboards, risk scores, and longitudinal trends confidently at the point of care [2, 11, 16]. The second is sustainability of digital competence over time. Many respondents described initial training that was useful at the moment of system rollout but not maintained [3, 9, 10]. Longitudinal work is needed to understand how competence is reinforced, how skills decay, and which training formats (for example microlearning during shift, simulation-based onboarding, peer mentoring) are most effective in real clinical environments [3, 6].

In summary, the study shows that healthcare professionals across Europe view digital competence as fundamental to care, but the conditions that enable that competence are unevenly distributed [3, 11, 14]. The main levers for improvement are not only individual upskilling but also protected time, interoperable infrastructure, structured support, and regionally equitable investment [3, 5, 21].

Overall, future research should move toward integrated, interdisciplinary approaches that not only assess competence gaps but also co-develop strategies for equitable digital health adoption across Europe [3, 19, 21, 23, 27].

## Conclusion

This study provides a comprehensive, cross-regional analysis of the digital competence of healthcare professionals across Europe, with a specific focus on the comparative dynamics between Northern and Southern countries. Through the integration of qualitative insights and quantitative data from over 2000 participants, it uncovers both the shared aspirations and the distinct realities shaping digital health adoption in these regions.

The findings reveal a persistent digital divide: Northern European countries—particularly Finland, Norway, and Denmark—demonstrate more advanced integration of digital tools in clinical settings, underpinned by strong institutional frameworks, continuous training programs, and mature digital infrastructures. National platforms like Finland’s Kanta system exemplify how strategic investments in interoperability and data governance can empower healthcare professionals to seamlessly incorporate electronic health records, telemedicine, and data-driven diagnostics into their workflows. In these contexts, digital competence is viewed not as an optional skill but as a core component of professional development and healthcare quality.

Conversely, in Southern Europe—most notably Portugal, Greece, and parts of Italy—the transition toward digital health remains hindered by systemic challenges. Healthcare professionals report facing fragmented systems, minimal exposure to structured training, limited institutional support, and infrastructural deficiencies, particularly in rural or underserved areas. These limitations are compounded by organizational cultures that may resist change and by time constraints that prevent professionals from engaging in digital upskilling. As a result, digital health tools are often perceived as burdensome rather than enabling, contributing to skepticism about their value and a slower pace of adoption.

Importantly, this North-South comparison underscores that digital transformation in healthcare is not merely a technological challenge but a deeply contextual and political one. Differences in national policies, funding mechanisms, and education systems profoundly influence the digital readiness of the healthcare workforce. While individual motivation and professional willingness to adopt digital tools exist across all regions, the structural conditions that enable such adoption vary dramatically.

This research contributes to the European discourse on digital health by highlighting that a one-size-fits-all approach to digital competence development is neither realistic nor effective. Tailored strategies are needed to address the specific barriers faced by healthcare professionals in different contexts. In Southern Europe, this means prioritizing investments in infrastructure, designing flexible training programs that accommodate high workloads, and implementing change management strategies that address cultural resistance. In Northern Europe, the focus should shift toward sustaining innovation, integrating emerging technologies such as AI and predictive analytics, and ensuring that digitalization continues to enhance rather than compromise the human aspects of care.

Ultimately, bridging the digital gap between North and South requires coordinated action at multiple levels—policy, institutional, and individual. Only through such alignment can we ensure that the promise of digital transformation is realized equitably across Europe, empowering all healthcare professionals to deliver safe, efficient, and patient-centered care in the digital age.

## Supplementary Information

Below is the link to the electronic supplementary material.

Supplementary Material 1

## Data Availability

The datasets used and/or analyzed during the current study are available from the corresponding author on reasonable request.
