# Supplementary material for: The imperative of digital competence in healthcare professionals: comparison between the North and South of Europe
Source: BMC Health Serv Res. 2026 Jan 10;26:191. doi: 10.1186/s12913-026-14000-8 (PMC12882289; doi:10.1186/s12913-026-14000-8)
Supplement: Supplementary file 1 — Supplementary Material 1 [file 12913_2026_14000_MOESM1_ESM.docx]

**Supplementary Appendix 1. Digital Competence in Healthcare: Self-Assessment Questionnaire**

Purpose:
This instrument was used to assess healthcare professionals’ experiences, competencies, and perceptions related to the use of digital tools in clinical practice. The questionnaire was structured according to the European Digital Competence Framework for Citizens (DigComp) and included five competence domains: information and data literacy, communication and collaboration, digital content creation, safety, and problem solving.

The survey contained four sections with 25 open-ended prompts (20 core items plus five contextual questions). Participants provided short narrative responses (2–5 sentences) based on personal experience.

Section A. Participant Background

1. What is your professional role (e.g., physician, nurse, radiographer, IT specialist, administrator)?
2. How many years of professional experience do you have in healthcare?
3. What is your primary workplace setting (e.g., hospital, primary care center, private practice, other)?
4. In which country and region do you currently work?
5. How would you describe the digital infrastructure of your organization (e.g., fully digital, partially digital, mostly paper-based)?

Section 1. Usage and Familiarity with Digital Tools

1. Which digital health tools (EHRs, telemedicine platforms, monitoring systems, scheduling software, etc.) do you use in your daily practice?
2. How frequently do you use electronic health records (EHRs) or similar documentation systems?
3. Are you familiar with telehealth or teleconsultation platforms? If so, how are they integrated into your workflow?
4. What types of digital tools do you consider essential for your work and why?
5. Have you experienced improvements in patient care, safety, or efficiency through digital tools? Please describe an example.

Section 2. Training Experiences and Needs

1. Have you received any formal training in digital health technologies or digital competence?
2. What training formats (e.g., workshops, online courses, peer learning, simulation) have you participated in?
3. Do you feel your current training is sufficient for the tools you use? Why or why not?
4. What digital competencies (skills or knowledge areas) do you think should be included in professional training for healthcare staff?
5. What kinds of support or resources (technical, educational, managerial) would help you improve your digital skills?
6. How often do you receive updates or refresher training when new systems are introduced?

Section 3. Perceived Barriers and Institutional Support

1. What are the main barriers you face when using digital tools (e.g., workload, technical issues, usability)?
2. Does your institution provide support for learning and integrating new digital technologies?
3. How do time constraints affect your ability to develop or practice digital skills?
4. Have you encountered resistance to change from colleagues or leadership regarding digital transformation?
5. What infrastructural or connectivity challenges (e.g., internet reliability, outdated equipment) do you experience in your setting?
6. How would you describe the availability of IT or technical support in your organization?

Section 4. Attitudes Toward Digital Transformation

1. How do you perceive the role of digital health in the future of healthcare delivery?
2. What is your opinion on the impact of digital tools on patient relationships and the quality of care?
3. How confident are you in interpreting data-driven information (dashboards, analytics, decision-support tools)?
4. What factors would motivate you to engage more deeply with digital health tools or training?
5. What actions can healthcare institutions and policymakers take to better prepare professionals for digital transformation?
6. Do you have any additional comments or reflections about digital competence in your professional context?

Administration Notes

Format: Online, open-ended questionnaire administered via AMR Educare
Estimated completion time: 20–30 minutes
Language: English
Target population: Physicians, nurses, radiographers, IT specialists, and departmental administrators working in hospital or primary-care settings
Inclusion criteria: Active use of at least one digital health tool in routine practice
Ethics approval: ISCTE School of Technologies and Architecture Ethics Committee (CE-ISTA/2025.04)
Data collection period: December 1, 2024 – January 15, 2025
